# SiO_2_‐CaO_CME_/Poly(Tetrahydrofuran)/Poly(Caprolactone) 3D‐Printed Scaffolds Drive Human‐Bone Marrow Stromal Cell Osteogenic Differentiation

**DOI:** 10.1002/adhm.202503733

**Published:** 2026-02-02

**Authors:** David R. Sory, Agathe C.M. Heyraud, Julian R. Jones, Sara M. Rankin

**Affiliations:** ^1^ National Heart and Lung Institute Imperial College London London UK; ^2^ Department of Materials Imperial College London London UK

**Keywords:** bioactive inorganic/organic hybrids, bone tissue engineering, human‐bone marrow stromal cells, osteogenic differentiation, sol‐gel

## Abstract

This article addresses the unmet clinical need of scaffolds for bone regeneration that can combine osteogenic properties, such as the promotion of bone marrow stem cell differentiation into osteoblasts, with the ability to withstand cyclic loading. In our previous study, we demonstrated that discs of SiO_2_‐CaO_CME_/poly(tetrahydrofuran)/poly(caprolactone) hybrids or their dissolution products can drive terminal osteogenic differentiation of human bone marrow stromal cells (h‐BMSCs) in vitro. The current study shows that the 3D‐printed hybrid scaffolds with physiologically relevant 3D architecture further promote h‐BMSC osteogenesis. The 3D‐printed scaffolds support spatially organized cell behavior in an environment mirroring conditions relevant to off‐the‐shelf implant applications. Primary cellular functions, including viability, adhesion, and proliferation, were maintained across 3D scaffold surfaces and within inter‐strut regions. osteogenic commitment was evidenced by the upregulation of lineage‐specific transcripts, hydroxyapatite deposition, and the organized assembly of extracellular matrix (ECM) proteins. Our results demonstrate that 3D‐printed scaffolds drive osteogenesis by modulating cell metabolism, inducing osteogenic morphological transitions, and promoting the expression of osteocalcin and collagen type I alpha 1 chain, alongside hydroxyapatite matrix mineralization. Collectively, our findings highlight the SiO_2_‐CaO_CME_/poly(tetrahydrofuran)/poly(caprolactone) scaffold's strong osteogenic properties—driven by composition, surface architecture, and ion release – and its promise for clinical bone regeneration.

## Graphical abstract

3D printed hybrid scaffolds combining bioactive silica–calcium chemistry with elastic polymers guide human bone stem cells to form bone. The scaffolds support cell survival, organization, and invasion while releasing osteogenic ions. Together, architecture and composition drive bone‐specific gene expression, extracellular matrix organization, and hydroxyapatite mineralization, highlighting strong promise for clinical bone regeneration.

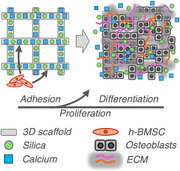

## 1 Introduction

In trauma surgery, there is an unmet clinical need for biomaterials that can regenerate large bone defects, where current surgical strategies of autograft or synthetic bone grafts are insufficient, termed ‘non‐union bone fractures’ [1]. Synthetic bone grafts that are commercially available are often based on calcium phosphates [2, 3] or bioactive glass [4]. Bioactive glass granules of the original 45S5 Bioglass composition were found to outperform synthetic hydroxyapatite granules of similar particle size in vivo, in terms of rate of bone ingrowth in a rabbit condyle model [5], which was attributed to the dissolution products of the glass having an osteogenic effect on osteoblasts [6]. The osteogenic effect of the dissolution products (calcium ions and silica species), later termed ‘osteostimulation’, was established through cell culture of osteoblasts in media that was preconditioned with the dissolution products of the melt‐derived 45S5 Bioglass particles (the particles were removed before culture) [7]. Interest in bioactive glasses has increased as they have been found to have antimicrobial properties, e.g., in the clinical treatment of osteomyelitis [8]. However, current synthetic bone grafts based on bioactive glass are only available in the form of granules or putties [9]. In addition to the osteostimulation and bone bonding properties, ideal scaffolds for bone regeneration should also have: an open interconnected pore network for vascularized bone ingrowth, with interconnected pore diameters in excess of 200 µm; mechanical properties that enable sharing of cyclic loads with the host bone, to enable mechanotransduction in the seeded cells; and controlled biodegradability [4]. Bioactive glass foams can be made with interconnected spherical pores that mimic cancellous bone [10, 11], or 3D‐printed into grid‐like (log‐pile‐style) scaffolds by direct ink‐writing [12, 13]. The 3D‐printed glass scaffolds can have high compressive strength (up to 150 MPa), with open pores larger than 100 µm, but they are brittle and cannot withstand cyclic loading. Recently, inorganic/organic sol‐gel hybrids have been developed that synergistically combine the properties of biodegradable polymers and bioactive glasses [14]. The hybrids are synthesized by introducing biodegradable polymers into the sol‐gel process.

The process for producing sol‐gel derived bioactive glasses usually involves the hydrolysis of silicate‐based alkoxides, such as tetraethoxysilane (TEOS), forming a sol (colloidal solution), from which a silicate network (gel) assembles by polycondensation of silanol bonds. To produce a bioactive glass composition, calcium is incorporated by adding calcium nitrate into the sol [15]. The gel must be heated to at least 450°C to allow calcium to diffuse into and act as a network modifier within the silicate structure [16].

We recently developed an inorganic/organic hybrid material with a unique combination of mechanical properties – including toughness, biodegradability, and 3D printability – composed of silica/poly(tetrahydrofuran)/poly(ε‐caprolactone), (SiO_2_/PTHF/PCL‐diCOOH) [14]. When 3D‐printed SiO_2_/PTHF/PCL‐diCOOH (20 wt.% SiO_2_) scaffolds with a pore channel width of ∼250 µm were seeded with h‐BMSCs and cultured in chondrogenic media, the stem cells underwent chondrogenic differentiation and produced articular cartilage ECM [17], which was enhanced under hypoxic conditions [18]. PCL scaffolds with similar pore size were used as a control, and the cells instead adopted a fibroblastic phenotype [17].

To impart bone bioactivity to the hybrid system, it was necessary to introduce Ca into the silica network so the inorganic network had a composition of SiO_2_‐CaO, with the aim of releasing Ca^2+^ ions during dissolution [19, 20]. Alkoxide precursors had to be used because processing temperatures needed to remain below 60°C and calcium addition increased compressive strength (17–64 MPa) and modulus of toughness (2.5–14 MPa) in bulk cylindrical samples [21]. Calcium methoxyethoxide (CME) was chosen as the calcium precursor to produce SiO_2_‐CaO_CME_/PTHF/PCL‐diCOOH hybrids, which has also been used in the synthesis of other hybrid materials [22, 23].

Sory et al. [24] investigated the osteogenic properties of 70S30C_CME_‐CL hybrid monoliths by exposing h‐BMSCs to media conditioned with the dissolution products of the hybrid discs for 21 days, assessing gene expression and ECM mineralization. They found that the conditioned media induced osteogenesis as effectively as osteogenic medium and promoted the deposition of hydroxyapatite in mineralized ECM. Osteogenic stimulation was evidenced by upregulated expression of osterix, and collagen type I alpha 1 chain after 4 days of culture and of integrin‐binding sialoprotein, alkaline phosphatase, osteocalcin, osteopontin, and matrix metalloproteinase 14 after 21 days. However, their study was restricted to dissolution products, without evaluating cell contact‐mediated osteogenic differentiation on 70S30C_CME_‐CL hybrid substrate, which would more accurately model its translational application.

Heyraud et al. determined that SiO_2_‐CaO_CME_/PTHF/PCL‐diCOOH with a TEOS: CME molar ratio of 70:30 (named 70S30C_CME_‐CL) could be 3D‐printed with a porosity of 50% and a yield strength of 0.90 ± 0.23 MPa and modulus of toughness 0.22 ± 0.04 MPa, compared to the Ca‐free SiO_2_/PTHF/PCL‐diCOOH hybrids (0.36 ± 0.14 MPa strength and 0.06 ± 0.01 MPa toughness modulus) [19]. They demonstrated that 70S30C_CME_‐CL scaffolds exhibit predominantly elastic behavior across physiological strain ranges and recover effectively from cyclic loading, confirming their mechanical stability under both static and dynamic conditions relevant to early‐stage trabecular bone repair.

This research focuses on a comprehensive evaluation of cell viability, adhesion, and metabolic activity, along with an analysis of the osteogenic potential of the SiO_2_‐CaO_CME_/PTHF/PCL‐diCOOH hybrids with h‐BMSCs cultured directly on either discs or 3D‐printed scaffolds of the 70S30C_CME_‐CL composition over 21 days. These properties were not examined in our previous studies. The central hypothesis is that the surface characteristics and ion release profile of 70S30C_CME_‐CL synergistically promote complete osteogenic differentiation of h‐BMSCs and ECM mineralization. To investigate osteogenesis, the study combines mRNA analysis of key transcription factors with protein assays and immunostaining to assess cell adhesion, matrix mineralization, and the production of bone‐specific ECM components.

## 2 Material and Methods

### 2.1 70S30C_CME_‐CL Hybrid Monolith and 3D Scaffold Manufacture

**FIGURE 1:**
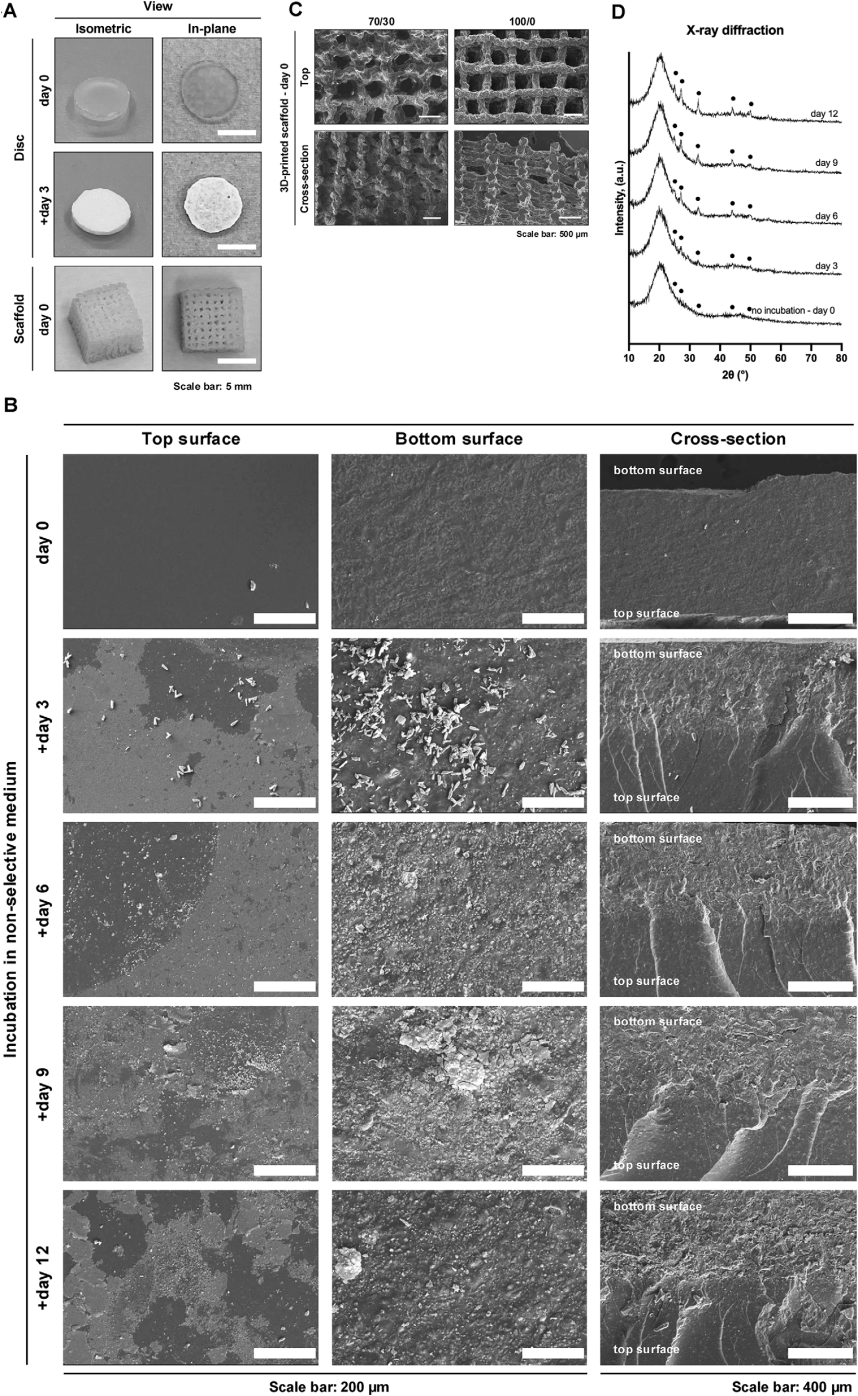
Manufacture and morphological analysis of 70S30C_CME_‐CL hybrid constructs. (A) Photographs of 8 mm diameter 70S30C_CME_‐CL discs (prior to and post incubation) and 8 mm (cubical) 3D‐printed scaffolds. (B) SEM micrographs of 70S30C_CME_‐CL (70/30) discs after 0, 3, 6, 9, and 12‐day incubation in non‐selective growth medium, followed by washing and drying. Images show the top and bottom surfaces, as well as cross‐sections (oriented with the bottom surface at the top of the image). Scale bar: 200 µm (top and bottom), 400 µm (cross section). (C) SEM micrographs of 70S30C_CME_‐CL and 100S0C_CME_‐CL (100/0) 3D‐printed scaffolds, showing the top surface and cross‐section. Scale bar: 500 µm. (D) X‐ray diffraction patterns of 70S30C_CME_‐CL monoliths at day 0 and after 3, 6, 9, and 12 days of incubation in serum‐free, non‐selective growth medium. Annotated peaks (●) indicate carbonate‐hydroxyapatite, as described by Legeros et al. [31] Data reproduced under terms of the CC‐BY license. [24] 2025, Sory et al., published by Biomaterials Advances.

70S30C_CME_‐CL hybrid discs and scaffolds (inorganic to organic, I:O, ratio of 25:75 wt.%, TEOS: CME 70:30, 30 mol.% calcium) were synthesized as described by Heyraud et al. [19]. Representative examples are shown in Figure 1a. Discs (8 mm diameter) were produced by pouring the sol into PTFE molds [24] and 3D‐printed scaffolds were produced using Direct Ink Writing, with a final dried pore size of 400–500 µm [19]. Both discs and scaffolds were sterilised by 3 washing cycles (15 min step^−1^) in deionised water (DI), followed by 100% ethanol, and then 70% ethanol, then irradiated under UV light for 3 h (turned halfway through), and finally air‐dried under sterile conditions for a minimum of 24 h. For comparison, calcium‐free 100S0C_CME_‐CL discs and scaffolds (I:O ratio 25:75 wt.%, 0 mol.% calcium) were produced and sterilized using the same protocol.

### 2.2 Scanning Electron Microscopy Imaging

Scanning electron microscopy (SEM) (JEOL 6010 LA) was carried out on 70S30C_CME_‐CL discs and 3D‐printed scaffolds in secondary electron mode at 20 kV with a working distance of 13–20 mm and a spot size of 40–60 µm. The samples were mounted on aluminum stubs using carbon adhesive tape, coated with a 10–15 nm gold layer using an EMITECH K575X Peltier‐cooled coater, and examined for top, bottom, and cross‐sectional views of sterilized 70S30C_CME_‐CL discs at baseline and after incubation for 3, 6, 9, and 12 days in serum‐free DMEM (11880‐028, Gibco). The incubation procedure followed protocols similar to those outlined in ISO 10993‐12 and ISO 10993–5, using 1 cm^2^ per monolith and a total surface area‐to‐volume ratio of 3 cm^2^ mL^−1^ (3 monoliths ml^−1^ of medium) [25, 26].

### 2.3 Cell Subculture

Fresh, unprocessed h‐BMSCs (PT2501, Lonza) from 3 donors were routinely subcultured using the Poietics MSCGM BulletKit (PT‐3001, Lonza) at 37°C in a humidified atmosphere with 5% CO_2_ (Table 1). Cells were detached using 0.05% Trypsin‐EDTA (Thermo Fisher Scientific). The h‐BMSCs were expanded in T225 cell culture flasks to approximately 90% confluency in α‐MEM‐based expansion medium. During testing, h‐BMSCs were cultured in either non‐selective growth medium (GM) or osteogenic medium (OM). All medium are detailed in Table S1. The h‐BMSC batches, and tri‐lineage differentiation potential (osteogenic, adipogenic, and chondrogenic) were identical to those reported in Sory et al. [24].

**TABLE 1:** Human‐bone marrow stromal cell batch and donor details.

Donor	Tissue acquisition No.	Lot No.	Sex	Age (y.o.)
1	31177	0000602009	M	23
2	31286	0000603525	M	37
3	32250	0000626977	M	22

### 2.4 Cell Seeding and Sample Preparation

Prior to cell seeding, all discs and 3D‐printed scaffolds (70S30C_CME_‐CL and 100S0C_CME_‐CL) were pre‐incubated for 72 h in serum‐free DMEM. h‐BMSCs were seeded onto the 8 mm diameter 70S30C_CME_‐CL and 100S0C_CME_‐CL hybrid discs or punched plastic coverslip controls (83.1840.002, non‐pyrogenic/endotoxin‐free, non‐cytotoxic, Sarstedt), and then transferred to fresh 48‐well untreated sterile tissue culture plates (TCPs) (677102, Greiner Bio‐One). Each sample received 40 µl of concentrated cell suspension (10^4^ cells disc^−1^) on the free‐surface of the disc (i.e., not mold‐facing) and was incubated for 2.5 h at 37°C, 5% CO_2_, and 21% O_2_ to allow cell attachment. Subsequently, 500 µl of fresh culture medium was added to each well.

For 3D‐printed scaffolds, h‐BMSCs were seeded using a Ficoll‐based culture medium as described by Cámara‐Torres et al. [27]. Ficoll, a high‐density, low‐viscosity aqueous solution containing sodium diatrizoate, was used to adjust cell sedimentation velocity, promoting scaffold surface contact and attachment. The seeding solution comprised 60% Ficoll‐Paque Plus (GE17‐1440‐02, Cytiva, Sigma–Aldrich) in FBS‐supplemented DMEM. Based on the adhesion rate observed in the disc model, two 30 µl droplets (65 750 cell ml^−1^) of the cell suspension were applied to each scaffold at 4 h intervals and incubated at 37°C, 5% CO_2_. After 24 h, cell‐seeded scaffolds were transferred to fresh 48‐well untreated sterile plates containing 500 µl of medium. In both disc and scaffold models, the medium was changed every other day starting on the day of seeding.

Cell cultures grown on coverslips are referred to as CoSlip and serve as the control group. h‐BMSCs cultured on 70S30C_CME_‐CL and 100S0C_CME_‐CL hybrid materials, whether on discs or on 3D‐printed scaffolds, are referred to as 70/30 and 100/0, respectively.

### 2.5 Total DNA Content

Total DNA content was quantified using the Quant‐iT PicoGreen dsDNA Assay Kit (PT11496, Invitrogen) according to the manufacturer's protocol. The assay determined the fraction of adherent cells relative to the initial cell seeding 24 h post‐seeding on hybrid discs and plastic coverslips. These substrates were chosen because they share the same size, seeding surface area, and relatively flat topography, allowing for direct and reliable comparison. Positive (adherent cells) and negative (non‐adherent cells) controls for cell adhesion consisted of h‐BMSCs seeded on culture‐treated and non‐adherent wells in TCP, respectively. After removing supernatant, cells were washed with PBS and lysed in 110 µl buffer (10 mm Tris, pH 8, 1 mm EDTA, 0.2% Triton X‐100, 0.1 mg ml^−1^ proteinase K), then incubated overnight at 4°C and at low speed on an orbital shaker. Lysates were homogenized and mixed 1:1 with PicoGreen dye, incubated for 5 min at room temperature in the dark. Fluorescence was measured using a SpectraMax i3x reader (Tecan, Molecular Devices, UK) at 480 ± 9 nm excitation and 520 ± 15 nm emission.

### 2.6 Cell Health and Proliferation

Health of h‐BMSCs was assessed over 14 days using the alamarBlue HS assay (DAL1025, Thermo Fisher Scientific) for discs and the CellTiter‐Glo 3D assay (G9681, Promega) for scaffolds, following the manufacturer's protocols. Fluorescence (alamarBlue) and luminescence (CellTiter‐Glo 3D) intensities from the dye–medium mixture served as indicators of cell health and proliferation. Measurements were taken on days 2, 4, 6, 10, and 14, with the untested days 0–2 allocated for cell seeding and attachment. At each timepoint, 100 µl of the medium was collected per well and analyzed using a SpectraMax I3x reader (Molecular Devices, UK) with SoftMax Pro 7 software. AlamarBlue fluorescence was measured in black‐walled, clear‐bottom 96‐well plates (3631, Corning) at 560 ± 15 nm excitation and 590 ± 9 nm emission; CellTiter‐Glo luminescence was recorded in opaque, white‐walled plates. h‐BMSCs cultured on coverslips in growth medium or growth medium containing polyurethane extract served as healthy and cytotoxic controls, respectively. Data were normalized to day 2 values to account for cell densities and enable comparison across models.

### 2.7 Mineralization Assay

After 21 days of treatment, the scaffolds were fixed in 4% (w/v) paraformaldehyde (28908, Thermo Fisher) in 2% FBS‐supplemented medium for 15 min at 37°C and 5% CO_2_, without agitation. Mineralization was assessed using the OsteoImage Assay (PA‐1503, Lonza), which detects calcium phosphate‐containing HA via fluorescence. After PBS washes, nuclei were counterstained with 10 µg ml^−1^ Hoechst 33342 (#33342, Thermo Fisher) for 15 min. Samples were mounted in µ‐Slide 8‐Well chambers (#80821, Ibidi) and imaged on a Leica SP8 inverted confocal microscope. Z‐stacks were acquired using sequential scanning with Kalman filtering, a 20× objective (NA 0.75), and 2048 × 2048 resolution. OsteoImage and Hoechst were imaged with 488/520 and 405 nm excitation, respectively. To minimize background fluorescence from scaffold‐deposited hydroxyapatite, imaging was focused on inter‐strut regions near the scaffold edges. Imaging was limited to 3D‐printed scaffolds, where their inter‐strut gaps reduced hybrid‐associated OsteoImage background, enabling the detection of cell‐derived mineralization.

### 2.8 Quantitative Real Time Polymerase Chain Reaction

Gene expression analysis was performed after 24 h, and on days 4, 7, 14, and 21 post‐seeding on both discs and 3D‐printed scaffolds. Total RNA was extracted using the RNeasy Mini Kit (Qiagen, 74106) following the manufacturer's instructions. RNA quantity and quality were assessed with a Nanodrop ND‐1000 spectrophotometer (Thermo Fisher Scientific). Reverse transcription was conducted in two steps: (1) Primer annealing: 0.125 µg of total RNA was mixed with 1 µl of 20 µg ml^−1^ Oligo(dT) primers (Promega, C1101) and RNase‐free water to a final volume of 35 µl. The mixture was incubated at 65°C for 5 min; (2) cDNA synthesis: The reaction was supplemented with 5 µl of 100 mm DTT, 5 µl of 10× AffinityScript RT buffer, 2 µl of 100 mm dNTPs, 2 µl of AffinityScript Reverse Transcriptase, and 1 µl of RNase block (all from Agilent Technologies). The final mix was incubated at 45°C for 1 h using a PTC‐200 thermocycler (MJ Research). Synthesized cDNA (50 µl) was stored at −20°C.

qPCR was performed on an Applied Biosystems ViiA 7 Real‐Time PCR System using 384‐well plates (Thermo Scientific, AB1384W). Each 10 µl reaction contained 4 µl cDNA, 5 µl Power SYBR Green Master Mix (Thermo Fisher Scientific, 4367659), and 1 µl of primer mix. Cycling conditions followed a standard three‐step protocol adapted from Ferreira et al. [28]: (1) AmpErase Uracil N‐glycosylase inactivation at 50°C for 2 min and denaturation at 95°C for 10 min; (2) annealing/extension at 58°C for 1 min; and (3) melting from 60°C to 95°C at 0.5°C min^−1^. Samples (*n* ≥ 4) were run in duplicate. No‐template controls (water instead of cDNA) were used to monitor for contamination.

Gene expression fold changes were calculated using the ΔΔCt method, normalized to the reference genes (RG) *Eukaryotic translation elongation factor 1 alpha 1* (*EEF1A1*) and *Ribosomal protein L13a* (*RPL13A*), and expressed relative to control h‐BMSCs cultured on a coverslip in GM at each time point (Table S2). Relative gene expression was calculated as: fold change in expression = 2^−ΔΔCt^, ΔΔCt = [treatment(CtGOI,t(x) − CtRG,t(x)) − control(CtGOI,t(x) − CtRG,t(x))] (GOI: gene of interest; RG: reference gene; t(x): timepoint) [29, 30]. The mRNA ratio of two genes was calculated as the relative change in their expression levels within the same sample. The ratio between two gene transcripts was also reported where relevant.

### 2.9 Osteocalcin Production

Osteocalcin levels were measured using the Human Osteocalcin DuoSet ELISA kit (DY1419, R&D Systems). Briefly, 96‐well plates were coated overnight at room temperature with 100 µl of capture antibody (844320) in PBS. After washing with buffer (WA126), wells were blocked with 300 µl of reagent diluent (DY004) for 1 h. Standards or samples (100 µl) were then added and incubated for 2 h, followed by a 2 h incubation with detection antibody (844321). After washing, 100 µl of streptavidin‐HRP (893975) was added for 20 min, then 100 µl of substrate solution (DY999) for 20 min in the dark. The reaction was stopped with 50 µl of stop solution (DY994), and absorbance was read at 450 nm with correction at 540 or 570 nm. Samples were run in triplicate, and osteocalcin concentrations were calculated from a standard curve (844322). Osteocalcin production (pg µg^−1^) in supernatant was measured after a 48 h Vitamin D3 treatment and normalized to total cellular protein.

### 2.10 Immunostaining

Twenty‐four hours post‐seeding, cell‐seeded discs and scaffolds were fixed with 4% paraformaldehyde for 20 min at room temperature and then permeabilized with 0.1% (v/v) Triton‐X 100 in PBS (PBT) for 10 min and blocked for 1 h with 10% (v/v) horse serum in 0.15% (w/v) glycine and 0.2% (w/v) bovine serum albumin (A2153, Sigma) in PBT. Samples were then incubated overnight at 4°C with 1:400 in PBT primary antibody Rat IgG2a monoclonal anti‐tubulin [YOL1/34] (#ab6161, Abcam). After 3 washes in phosphate buffered saline (PBS, without calcium and magnesium), h‐BMSCs were incubated in the dark for 1 h at room temperature with 1:300 Alexa Fluor 568 goat anti‐rat (#ab175476, Abcam). Negative controls (omission of the primary antibody with the presence of secondary antibodies) were performed. No staining was observed in the samples used as negative controls. Other h‐BMSCs were stained for 1 h at room temperature in the dark with 1:100 Phalloidin‐TRITC (P1951, Sigma). Nuclei were counterstained with 10 µg ml^−1^ Hoechst Trihydrochloride Trihydrate for 15 min. The stained hybrids were imaged on a Leica SP8 inverted confocal laser scanning microscope. Detector gains were set to be constant between samples to facilitate comparison. Z‐series were obtained using sequential acquisition and Kalman filter mode, 10× or 20× objective with numerical aperture of 0.75, and 2048 × 2048 pixel size. Images for tubulin and actin are maximum intensity projections of at least 20 Z‐slices of ≤0.5 µm obtained using the Image J.

### 2.11 Statistical Analyses

All results are reported as mean ± standard deviation (SD) of at least three independent experiments based on 3 donors (*N* ≥ 3), unless otherwise stated. The samples were prepared at least in quadruplet (*n* ≥ 4) (for each loading condition and for each independent experiment). Prior to statistical testing, outliers were identified using Grubbs’ test (α = 0.05), and normality was assessed using the Shapiro–Wilk test (α = 0.05). Upon exclusion of outliers and normality validation, statistical analyses were carried out by using ordinary one‐way or two‐way ANOVAs with Tukey's multiple comparison tests to detect statistical significance between conditions. Otherwise, non‐parametric Kruskal–Wallis tests with Dunn's multiple comparison tests were used to detect statistical significance between conditions. Plots show individual technical replicates and average data expressed as mean ± SD. Gene expression levels are shown as fold change normalized to expression in h‐BMSCs cultured with basal medium control at the same time point (CoSlip). The coefficient of variation was used to compare the scatter of variables. It is computed as the standard deviation divided by the mean and is expressed as a percentage. Statistical analyses were performed using GraphPad Prism software (version 9.1.1 on MacOS, San Diego, USA). *p‐*values are indicated in figure captions.

### 2.12 Nomenclature

Protein and gene symbols use the same abbreviation. Protein designations are written in non‐italicized uppercase letters. Gene symbols are written in italicized uppercase letters. Messenger RNAs follow the same formatting conventions as gene symbols.

## 3 Results and Discussion

### 3.1 Bioactive 70S30C_CME_‐CL Hybrids Promote Surface Mineralization and Hydroxyapatite Deposition

The 100S0C_CME_‐CL and 70S30C_CME_‐CL hybrid discs and 3D‐printed scaffolds are shown in Figure 1a and are referred to as 100/0 and 70/30, respectively, throughout the figures. Both the 70S30C_CME_‐CL disc and 3D‐printed scaffold hybrids formed hydroxycarbonate apatite (HCA), a component of the bone matrix, on their surfaces over four sequential incubation periods in DMEM, with progressive increases in deposition every three days (Figure 1b–d). SEM analysis of disc cross‐sections revealed incubation‐induced degradation progressing from the surface toward the interior of the disc. The PTFE mold‐facing surface of the disc supported better nucleation and HCA formation, compared to the smoother air‐facing surface. Prolonged immersion resulted in more visible deposition, even on the smoother regions. Our SEM observations are consistent with X‐ray diffraction analyses performed in our dissolution study on hybrid monoliths [24], which confirmed HCA deposition after incubation in medium compared to non‐incubated controls (Figure 1c). Characteristic peaks in the XRD patterns at 25°, 27°, 32°, 44°, and 49° 2*θ* matched the HA standard pattern described by Legeros et al. [31].

### 3.2 Bioactive 70S30C_CME_‐CL Hybrids Support h‐BMSC Adhesion, Proliferation, and Cytoskeletal Organization

**FIGURE 2:**
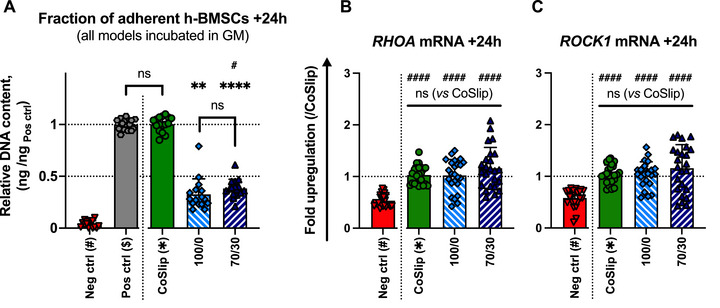
Adhesion of h‐BMSCs on 70S30C_CME_‐CL discs in vitro. (A) Fraction of adherent h‐BMSCs on 70S30C_CME_‐CL (70/30) discs, cell‐treated coverslips (CoSlip), and 100S0C_CME_‐CL (100/0) discs (*n* ≥ 8, *N* = 3). Culture‐treated and non‐adherent TCP wells served as positive and negative controls, respectively. Gene expression of (B) *RHOA* and (C) *ROCK1* mRNAs was assessed after a 24‐h culture on 70S30C_CME_‐CL, CoSlip, and 100S0C_CME_‐CL discs (*n* ≥ 4, *N* = 3). Statistical significance was determined using a Kruskal‐Wallis test with Dunn's post‐hoc comparison. * and # indicate significance relative to CoSlip and TCP controls, respectively. ns: *p* > 0.05, ^*^ *p* ≤ 0.05, ^**^ *p* < 0.01, ^***^ *p* < 0.001, ^****^ *p* < 0.0001.

Total DNA quantification revealed that 38.3 ± 8.8% of seeded h‐BMSCs adhered to 70S30C_CME_‐CL discs, and 32.5 ± 15.2% adhered to 100S0C_CME_‐CL discs within 24 h (Figure 2a). HCA deposition on the 70S30C_CME_‐CL surface modestly increased adhesion by 5.8 ± 17.6%, although the difference between substrates was not statistically significant. The CoSlip group achieved 100.4 ± 7.2% adhesion, with ∼90% confluency after 24 h, whereas non‐adherent surfaces resulted in only 4.0 ± 3.2% h‐BMSC adhesion as expected for non‐adherent surfaces.

RhoA and its downstream effector ROCK1 regulate cell adhesion, motility, and focal adhesion formation. Analysis of *RHOA* and *ROCK1* mRNA expression in adherent h‐BMSCs showed no significant differences between CoSlip, 100S0C_CME_‐CL, and 70S30C_CME_‐CL surfaces (Figure 2b,c). However, both hybrid surfaces exhibited greater dispersion than CoSlip control groups (coefficient of variation of *RHOA* mRNA: CoSlip = 17.70%, 100S0C_CME_‐CL = 30.34%, 70S30C_CME_‐CL = 34.59%; and of *ROCK1* mRNA: CoSlip = 17.43%, 100S0C_CME_‐CL = 23.70%, 70S30C_CME_‐CL = 39.35%). In some samples, hybrid surfaces induced up to a two‐fold increase in adhesion marker expression compared to CoSlip controls, whereas other samples exhibited downregulation, to levels approaching those observed in non‐adherent controls.

**FIGURE 3:**
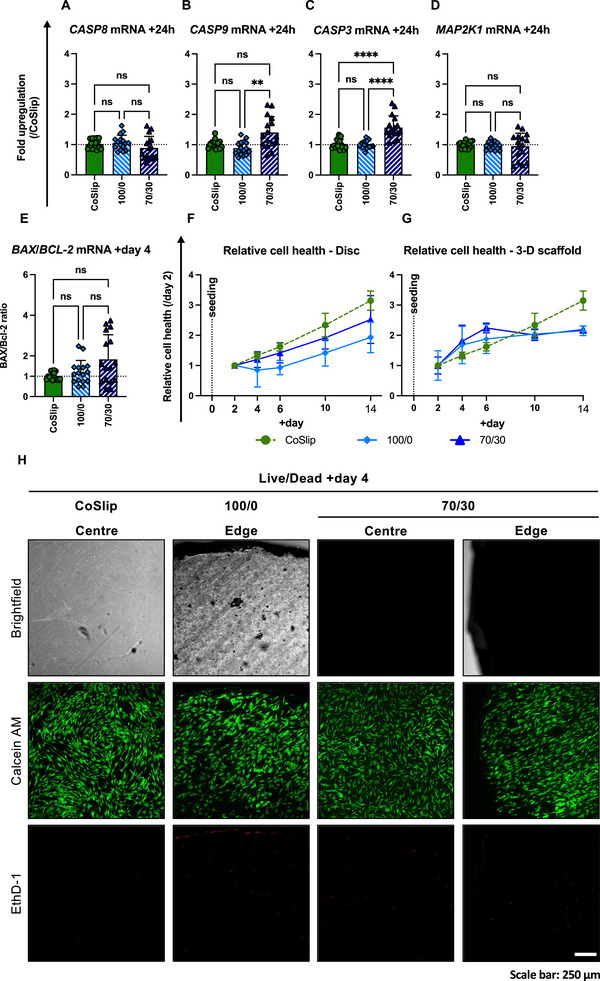
Early survival and long‐term cell health in h‐BMSCs cultured on 70S30C_CME_‐CL discs and 3D‐printed scaffold in vitro. (A–F) Gene expression analyses for (A) *CASP8*, (B) *CASP9*, (C) *CASP3* and (D) *MAP2K1* mRNAs in h‐BMSCs cultured for 24 h on 70S30C_CME_‐CL (70/30) and 100S0C_CME_‐CL (100/0) discs. (*n* ≥ 8, *N* ≥2). (E) *BAX/BCL‐2* mRNA ratio in h‐BMSCs cultured for 4 days on 70S30C_CME_‐CL and 100S0C_CME_‐CL discs (*n* ≥ 8, *N* ≥2). (F‐G) Relative cell health in h‐BMSCs cultured for 12 days on 70S30C_CME_‐CL and 100S0C_CME_‐CL (F) discs and (G) 3D‐printed scaffolds. Statistical significance was determined using a Kruskal‐Wallis test with Dunn's post‐hoc comparison. ns: *p* > 0.05, ^*^ *p* ≤ 0.05, ^**^ *p* < 0.01, ^***^ *p* < 0.001, ^****^ *p* < 0.0001. (H) Representative confocal micrographs from a Live/Dead assay 48 h post‐seeding on 70S30C_CME_‐CL and 100S0C_CME_‐CL discs. CoSlip refers to h‐BMSCs cultured on tissue culture‐treated coverslips. Calcein AM (green) marks viable cells, while EthD‐1 (red) indicates dead cells in monochrome images. Images were acquired on a Leica TCS SP5 MP/FLIM inverted confocal microscope (Leica Microsystems, UK). Scale bar: 250 µm.

No upregulation of *CASP8*, *CASP9*, *MAP2K1* mRNAs, or in the *BAX/BCL‐2* mRNA ratio, was observed in h‐BMSCs seeded on either hybrid relative to CoSlip controls (Figure 3a,b,d). However, *BAX/BCL‐2* mRNA ratios showed greater variation (coefficient of variation: 70S30C_CME_‐CL = 66.14%), and *CASP3* mRNA expression was elevated in h‐BMSCs seeded on 70S30C_CME_‐CL compared to CoSlip controls (Figure 3c,e). Despite indications of potential apoptotic processes, Live/Dead assays at 48 h confirmed h‐BMSC attachment and survival on the hybrid surface with minimal cell death observed at that time point (Figure 3h).

We found that culture substrate format (disc or 3D‐printed scaffold), calcium availability (70S30C_CME_‐CL vs 100S0C_CME_‐CL), and exposure duration were key determinants of h‐BMSC health and proliferation (Figure 3f,g). After 48 h, h‐BMSCs proliferated steadily on both 100S0C_CME_‐CL and 70S30C_CME_‐CL discs, although proliferation on 100S0C_CME_‐CL discs decreased slightly until day 6, after which it recovered to levels similar to those on 70S30C_CME_‐CL. Both hybrid materials exhibited lower cell health compared to CoSlip controls. On scaffolds, h‐BMSC proliferation increased more rapidly than CoSlip controls during the 2–6 day period, but slowed between days 6 and 14, stabilizing at approximately double the 48 h cell health value.

**FIGURE 4:**
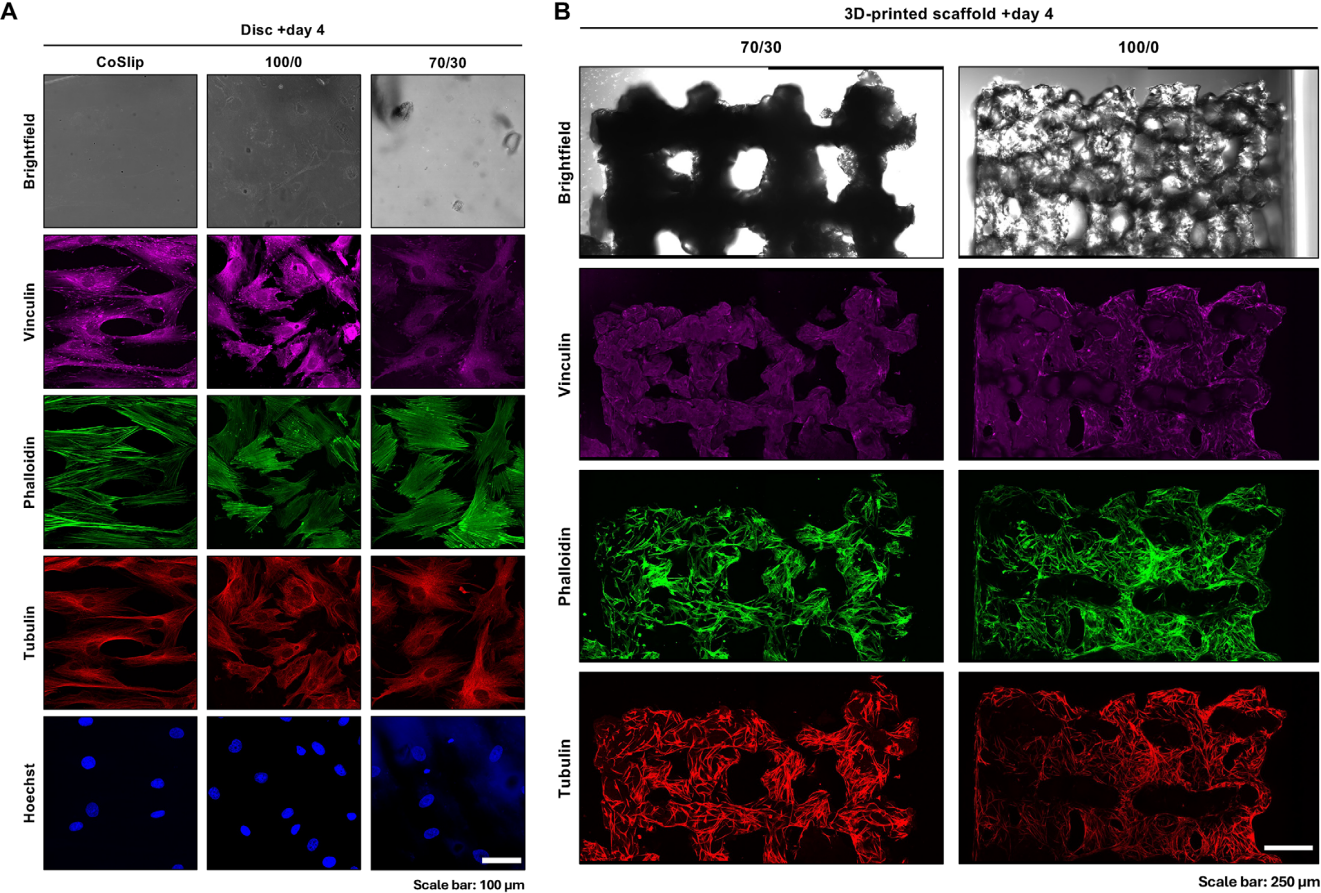
Adhesion of h‐BMSCs on 70S30C_CME_‐CL discs and 3D‐printed scaffolds in vitro. (A) Representative confocal micrographs of h‐BMSCs at day 4 post‐seeding on 70S30C_CME_‐CL (70/30) and 100S0C_CME_‐CL (100/0) discs. Vinculin (purple) marks focal adhesions, tubulin (red) highlights the microtubule network, phalloidin (green) stains F‐actin, and Hoechst (blue) labels cell nuclei. Images were acquired using a Leica TCS SP5 MP/FLIM confocal microscope (Leica Microsystems, UK). Scale bar: 250 µm. (B) Representative confocal micrographs of h‐BMSCs at day 4 post‐seeding on 70S30C_CME_‐CL and 100S0C_CME_‐CL 3D‐printed scaffolds. Same staining and imaging conditions as panel A. Scale bar: 250 µm.

By day 4, h‐BMSCs exhibited well‐organized cytoskeletal structures and distinct focal adhesion distribution across all samples. Vinculin staining revealed abundant, well‐developed focal adhesions at the cell periphery, indicating effective cell spreading and strong cell‐surface interactions (Figure 4a,b). Phalloidin staining suggested denser actin‐filament networks in h‐BMSCs on 100S0C_CME_‐CL and 70S30C_CME_‐CL discs, while cells on coverslips displayed prominent stress fibers spanning the entire cell body. Our results suggest substrate‐dependent differences in cytoskeletal organization between the hybrid discs, which have uneven surfaces, and the smoother polystyrene coverslips.

### 3.3 Bioactive 70S30C_CME_‐CL Hybrids Promote Osteoblast Differentiation via Gene Activation

We previously demonstrated that continuous exposure to 70S30C_CME_‐CL dissolution products (72 h incubation) induces osteogenic differentiation of h‐BMSCs over 21 days on standard TCPs [24] and promotes hydroxyapatite‐enriched ECM synthesis. Consistent with those findings, a time‐course analysis of key markers demonstrated robust osteogenic stimulation in h‐BMSCs cultured directly on 70S30C_CME_‐CL 3D‐printed scaffolds for 21 days.

**FIGURE 5:**
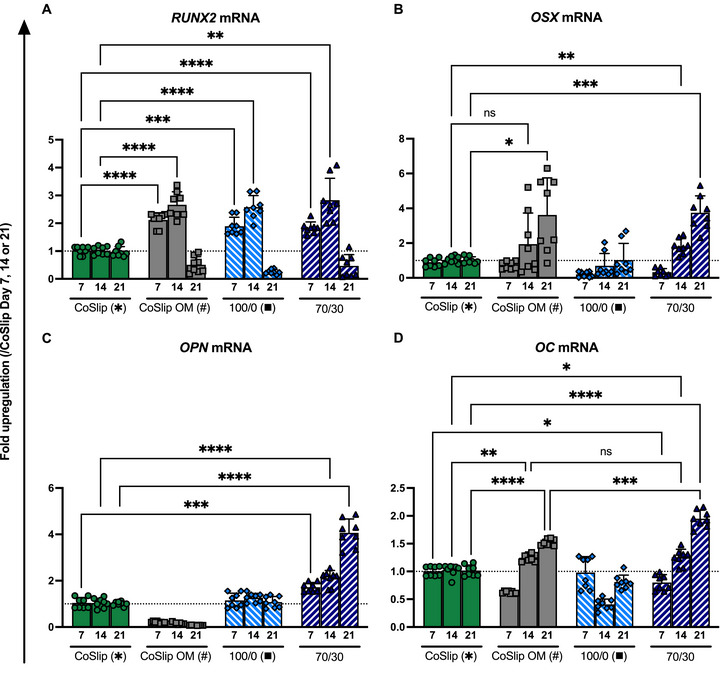
Gene expression profiles of early osteogenic markers in h‐BMSCs cultured on 70S30C_CME_‐CL 3D‐printed scaffolds in vitro. (A–D) Gene expression analyses for (A) *RUNX2*, (B) *OSX*, (C) *OPN*, (D) *OC* mRNAs in h‐BMSCs cultured for 7, 14 and 21 days on coverslips CoSlip, 100S0C_CME_‐CL (100/0) and 70S30C_CME_‐CL (70/30) 3D‐printed scaffolds (*n* ≥ 4, *N* ≥ 3). CoSlip and CoSlip OM represent control samples with h‐BMSCs cultured on culture‐treated coverslips in non‐selective and osteogenic induction media (OM), respectively. Expression levels are presented as fold changes normalized to the expression levels of the CoSlip control. Data are shown as individual technical replicates and mean ± SD. Statistical significance was determined using a non‐parametric Kruskal‐Wallis test with Dunn's multiple comparisons. * and # indicate significance relative to the CoSlip and CoSlip OM controls, respectively. ns: *p* > 0.05, ^*^ *p* ≤ 0.05, ^**^ *p* < 0.01, ^***^ *p* < 0.001, ^****^ *p* < 0.0001.

RUNX2, a DNA‐binding transcription factor containing a Runt domain, serves as a master regulator of early osteoblast differentiation [32]. In the 3D‐printed scaffold experiments, *RUNX2* mRNA expression displayed similar patterns on both 100S0C_CME_‐CL and 70S30C_CME_‐CL scaffolds (Figure 5a). Expression levels on days 7 and 14 were higher than basal media and similar to OM CoSlip controls in which osteogenesis was chemically induced, confirming that both scaffolds support early osteogenic commitment. Consistent with in vivo upregulation during early osteogenesis and suppression during maturation [32, 33], RUNX2 was significantly downregulated below baseline at the later stage (day 21) in OM‐treated cells and both 100S0C_CME_‐CL and 70S30C_CME_‐CL groups.

Osterix (OSX), a transcription factor essential for pre‐osteoblast maturation, regulates osteogenic differentiation of h‐BMSCs in both RUNX2‐dependent and RUNX2‐independent pathways [34, 35, 36]. In RUNX2‐dependent pathways, OSX functions downstream to mediate osteogenic signaling [34, 35]. We found that *OSX* mRNA expression was significantly increased at day 14, peaking at day 21 in h‐BMSCs cultured on 70S30C_CME_‐CL hybrid scaffolds, compared to CoSlip and CoSlip OM controls (Figure 5b). No significant upregulation was observed at day 7, and OSX expression remained low or undetectable across all time points in the calcium‐free 100S0C_CME_‐CL groups.

RUNX2 primes h‐BMSCs for osteoblast differentiation by interacting with transcriptional co‐activators, such as OSX, to drive the expression of key osteogenic genes, including osteocalcin (OC) and collagen type I alpha 1 chain (COL1A1) [34, 37, 38, 39]. While the expression of *RUNX2* and *OSX* mRNA is initially necessary, it alone is insufficient to demonstrate osteogenic priming in h‐BMSCs. The presence of the corresponding transcription factor proteins, along with the production and activity of downstream effectors, such as OC and COL1A1, is required to progress osteogenesis. Gene upregulation in the 70S30C_CME_‐CL hybrid 3D scaffold group was comparable to that observed under chemically induced osteogenesis, confirming the scaffold capacity to effectively stimulate early pathways involved in osteogenic differentiation.

Osteopontin (OPN), a phosphorylated glycoprotein, regulates cell adhesion, lineage commitment, and biomineralization during osteogenesis [40]. In this study, *OPN* mRNA was upregulated by day 7 and peaked at day 21. In contrast, expression remained low and unchanged over time in OM‐treated CoSlip and 100S0C_CME_‐CL groups (Figure 5c).

OC is a key osteogenic marker and the second most abundant osteoid protein after COL1A1 [41]. On days 14 and 21, *OC* mRNA was significantly upregulated in h‐BMSCs cultured on 3D‐printed 70S30C_CME_‐CL scaffolds and in CoSlip OM controls but was downregulated at day 7 (Figure 5d). In contrast, *OC* mRNA levels remained unchanged or were downregulated in 100S0C_CME_‐CL hybrids.

**FIGURE 6:**
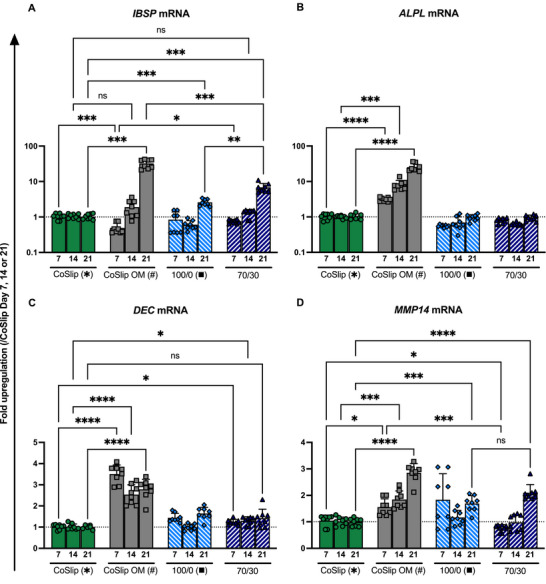
Gene expression profiles of late osteogenic markers in h‐BMSCs cultured on 70S30C_CME_‐CL 3D‐printed scaffolds in vitro. (A–D) Gene expression analyses for (A) *IBSP*, (B) *ALPL*, (C) *DEC* and (D) *MMP14* mRNAs in h‐BMSCs cultured for 7, 14 and 21 days on coverslips CoSlip, 100S0C_CME_‐CL (100/0) and 70S30C_CME_‐CL (70/30) 3D‐printed scaffolds (*n* ≥ 4, *N* ≥ 3). CoSlip and CoSlip OM represent control samples with h‐BMSCs cultured on culture‐treated coverslips in non‐selective and osteogenic induction media (OM), respectively. Expression levels are presented as fold changes normalized to the expression levels of the CoSlip control. Data are shown as individual technical replicates and mean ± SD. Statistical significance was determined using a non‐parametric Kruskal‐Wallis test with Dunn's multiple comparisons. * and # indicate significance relative to the CoSlip and CoSlip OM controls, respectively. ns: *p* > 0.05, ^*^ *p* ≤ 0.05, ^**^ *p* < 0.01, ^***^ *p* < 0.001, ^****^ *p* < 0.0001.

Integrin‐binding sialoprotein (IBSP) and alkaline phosphatase (ALPL) are key regulators of osteogenesis, supporting matrix mineralization and phosphate hydrolysis, respectively. *IBSP* mRNA was significantly upregulated at day 21 across all models compared to CoSlip controls (*p* ≤ 0.019) and day 14 within the same groups (*p* ≤ 0.0002), while remaining unchanged or downregulated before day 14 (Figure 6a).

**FIGURE 7:**
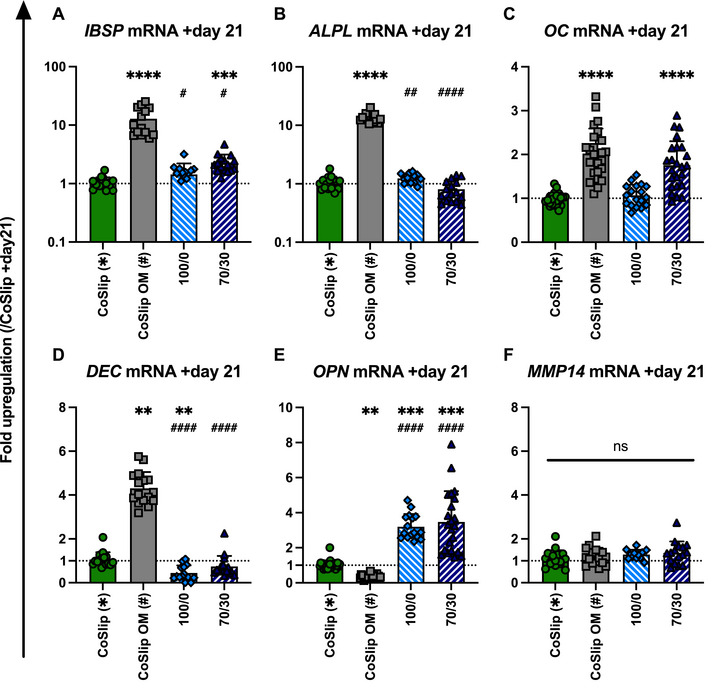
Gene expression profiles of late osteogenic markers in h‐BMSCs cultured on 70S30C_CME_‐CL discs in vitro. (A–F) Gene expression of (A) *IBSP*, (B) *ALPL*, (C) *OC*, (D) *DEC*, (E) *OPN* and (F) *MMP14* mRNAs in h‐BMSCs cultured for 21 days on 100S0C_CME_‐CL (100/0) and 70S30C_CME_‐CL (70/30) discs (*n* ≥ 4, *N* ≥ 3). CoSlip and CoSlip OM represent cells cultured on coverslips in non‐selective and osteogenic media (OM), respectively. Data are shown as fold changes normalized to CoSlip, with individual technical replicates and mean ± SD. Statistical significance was determined using a Kruskal‐Wallis test with Dunn's post‐hoc comparison. * and # indicate significance relative to CoSlip and CoSlip OM controls, respectively. ns: *p* > 0.05, ^*^ *p* ≤ 0.05, ^**^ *p* < 0.01, ^***^ *p* < 0.001, ^****^ *p* < 0.0001.

*ALPL* mRNA was consistently upregulated in CoSlip OM samples across all time points (days 7, 14, and 21), with no significant changes observed in 100S0C_CME_‐CL or 70S30C_CME_‐CL groups regardless of calcium presence (Figure 6b). Only the OM groups upregulated DEC mRNA levels compared to CoSlip controls at each time point (Figure 6c). Similarly, only the OM groups upregulated MMP14 mRNA compared to CoSlip controls, with the exception that the 70S30CCME‐CL hybrid significantly upregulated this mRNA marker on day 21 (Figure 6d). The comparison with the 2D disc model revealed that *OPN*, *OC*, *IBSP*, and *ALPL* mRNA expression patterns in h‐BMSCs on 70S30C_CME_‐CL discs closely matched those measured on 3D‐printed scaffolds at day 21 (Figure 7a–f; Table S3).

### 3.4 Bioactive 70S30C_CME_‐CL Hybrids Drive h‐BMSCs to Osteoblasts and Stimulate the Synthesis of Extracellular Matrix Components

**FIGURE 8:**
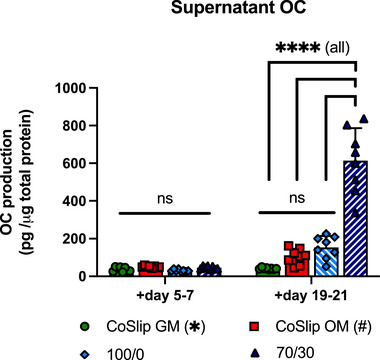
Osteocalcin production by h‐BMSCs on 70S30C_CME_‐CL 3D‐printed scaffold in vitro. Osteocalcin (OC) protein levels in supernatant for day periods 5–7 and 19–21 from h‐BMSCs cultured on 70S30C_CME_‐CL (70/30), 100S0C_CME_‐CL (100/0) 3D‐printed scaffolds (*n* ≥ 4, *N* ≥ 2). CoSlip and CoSlip OM represent cells cultured on coverslips in non‐selective and osteogenic media, respectively. Statistical significance was determined using a Kruskal‐Wallis test with Dunn's post‐hoc comparison. ns: *p* > 0.05, ^*^ *p* ≤ 0.05, ^**^ *p* < 0.01, ^***^ *p* < 0.001, ^****^ *p* < 0.0001.

The transition of osteoprogenitors into fully mature osteoblasts is indicated by the production of bone matrix proteins such as COL1A1 and OC, along with other ECM components [42, 43]. We found a time‐dependent increase in OC release in the supernatant, consistent with the *OC* mRNA results. OC production was minimal across all groups on day 7, but by day 21, levels significantly increased in h‐BMSCs cultured on 70S30C_CME_‐CL scaffolds (613 ± 172.5 pg µg^−1^, *p*‐value < 0.0001 vs. all other groups) compared to lower production on 100S0C_CME_‐CL scaffolds and control groups (ns, *p* ≥ 0.1) (Figure 8). Klingelhöffer et al. also observed lower OC levels in OM groups, suggesting that osteogenic induction medium suppresses OC transcription [44]. Our findings strongly indicated that 70S30C_CME_‐CL hybrids promote late‐stage osteoblast differentiation and synthesis of a key component of bone ECM.

**FIGURE 9:**
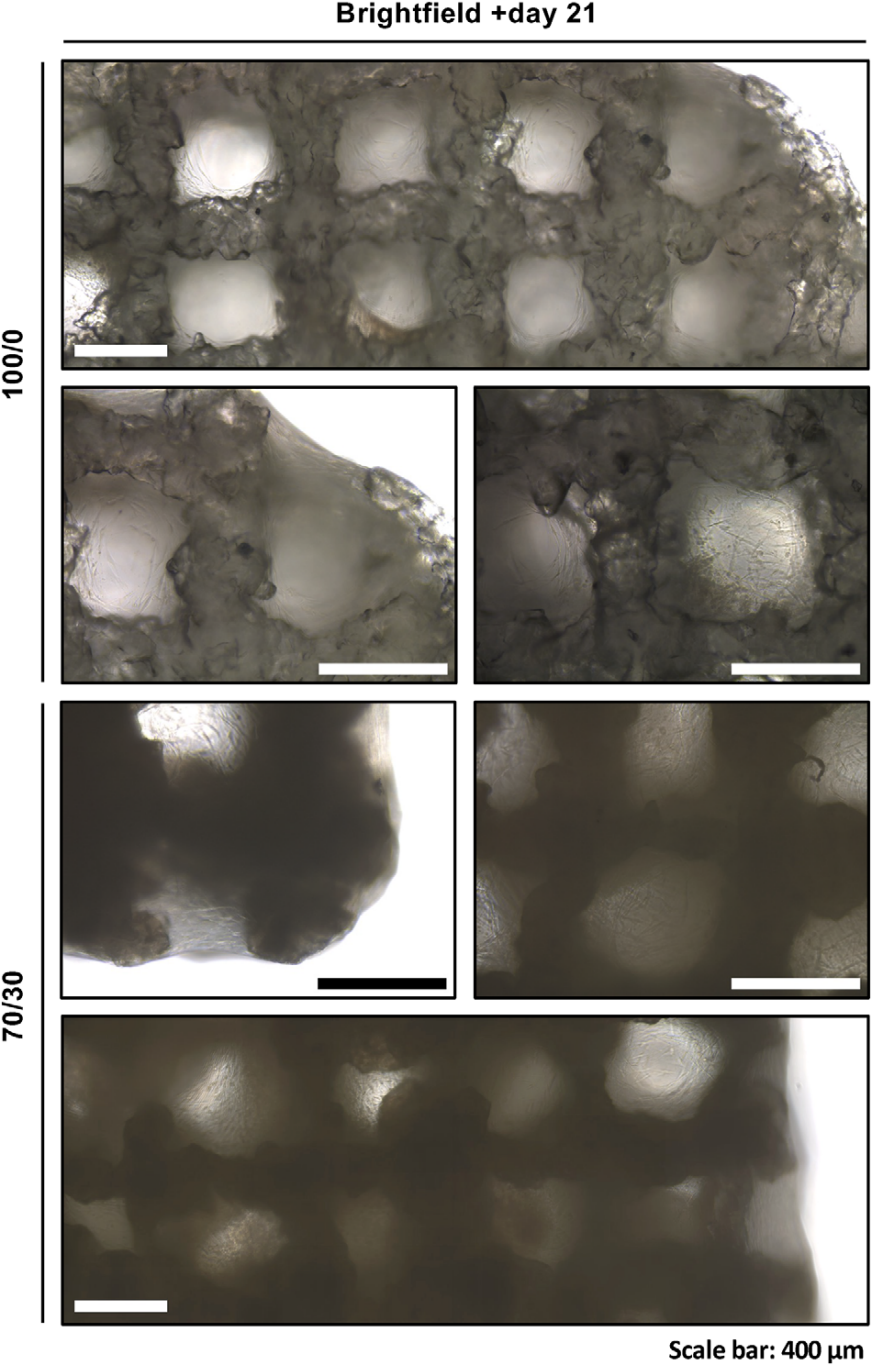
Production of extracellular matrix‐like fibrous structures on 100S0C_CME_‐CL and 70S30C_CME_‐CL 3D‐printed scaffolds in vitro. Representative micrographs in brightfield of 100S0C_CME_‐CL (100/0) and 70S30C_CME_‐CL (70/30) scaffolds at day 21 post‐seeding. Scale bar: 400 µm.

**FIGURE 10:**
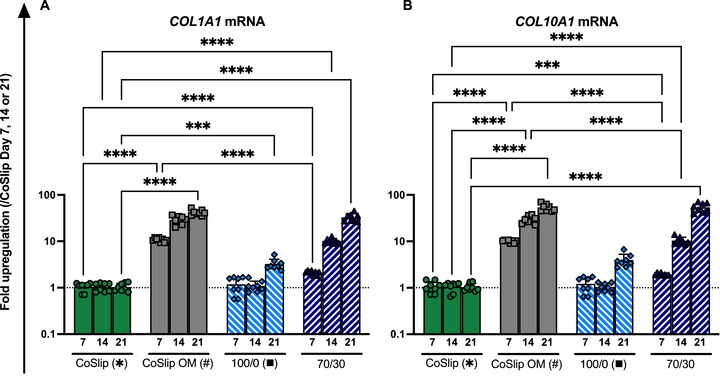
Gene expression profiles of collagen markers in h‐BMSCs cultured on 70S30C_CME_‐CL 3D‐printed scaffolds in vitro. (A‐B) Gene expression analyses for (A) *COL1A1*, (B) *COL10A1* mRNAs in h‐BMSCs cultured for 7, 14 and 21 days on coverslips CoSlip, 100S0C_CME_‐CL (100/0) and 70S30C_CME_‐CL (70/30) 3D‐printed scaffolds (*n* ≥ 4, *N* ≥ 3). CoSlip and CoSlip OM represent control samples with h‐BMSCs cultured on culture‐treated coverslips in non‐selective and osteogenic induction media, respectively. Expression levels are presented as fold changes normalized to the expression levels of the CoSlip control. Data are shown as individual technical replicates and mean ± SD. Statistical significance was determined using a non‐parametric Kruskal‐Wallis test with Dunn's multiple comparisons. * and # indicate significance relative to the CoSlip and CoSlip OM controls, respectively. ns: *p* > 0.05, ^*^ *p* ≤ 0.05, ^**^ *p* < 0.01, ^***^ *p* < 0.001, ^****^ *p* < 0.0001.

**FIGURE 11:**
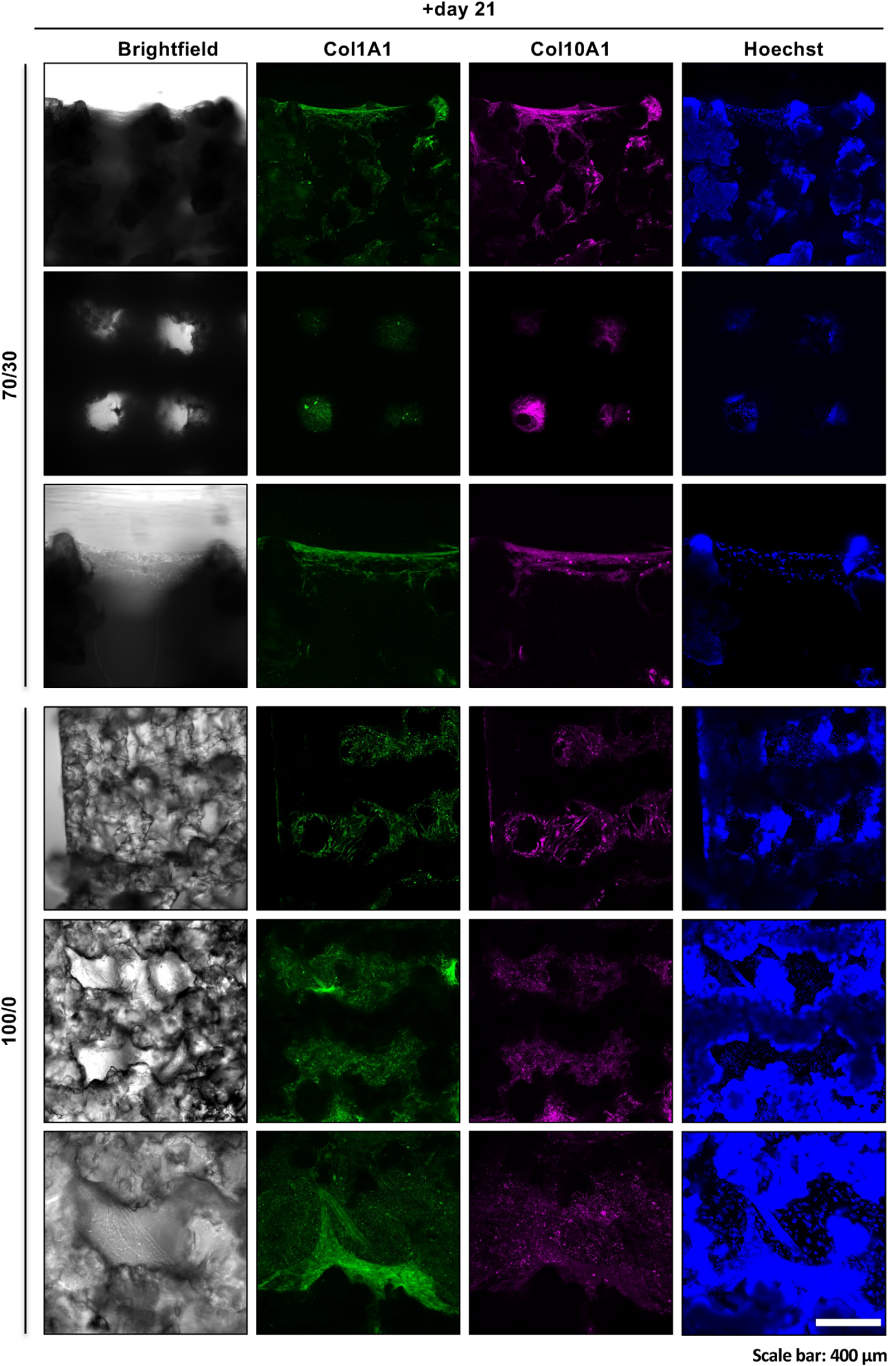
Collagen‐enriched extracellular matrix on 70S30C_CME_‐CL 3D‐printed scaffolds in vitro. Representative confocal micrographs showing the enrichment in collagen type I alpha 1 (COL1A1) and collagen type X alpha 1(COL10A1) in the extracellular matrix of 70S30C_CME_‐CL and 100S0C_CME_‐CL scaffolds. Hoechst (blue) labels h‐BMSC nuclei and creates background fluorescence due to possible scaffold retention or adsorption of Hoechst in the hybrid matrix. Scale bar: 250 µm.

During continuous monitoring up to day 21, brightfield microscopy revealed the development of ECM‐like fibrous structures within the pore spaces of both the 100S0C_CME_‐CL and 70S30C_CME_‐CL 3D‐printed scaffolds (Figure 9). At the same time point, transcriptomic analyses showed increased expression of *COL1A1* and *COL10A1* mRNAs in the 70S30C_CME_‐CL groups and CoSlip OM controls (Figure 10). COL1A1 is abundantly secreted by mature osteoblasts in bone tissue [45]. Immunohistochemistry further indicated the presence of COL1A1 and COL10A1 proteins within the scaffolds, supporting the transcriptomic findings. Hoechst staining revealed adherent cells on and between scaffold struts, with progressive ECM formation filling the pores (Figure 11). The presence of COL1A1 and OC strongly suggests active RUNX2 and OSX transcriptional activity in h‐BMSCs.

**FIGURE 12:**
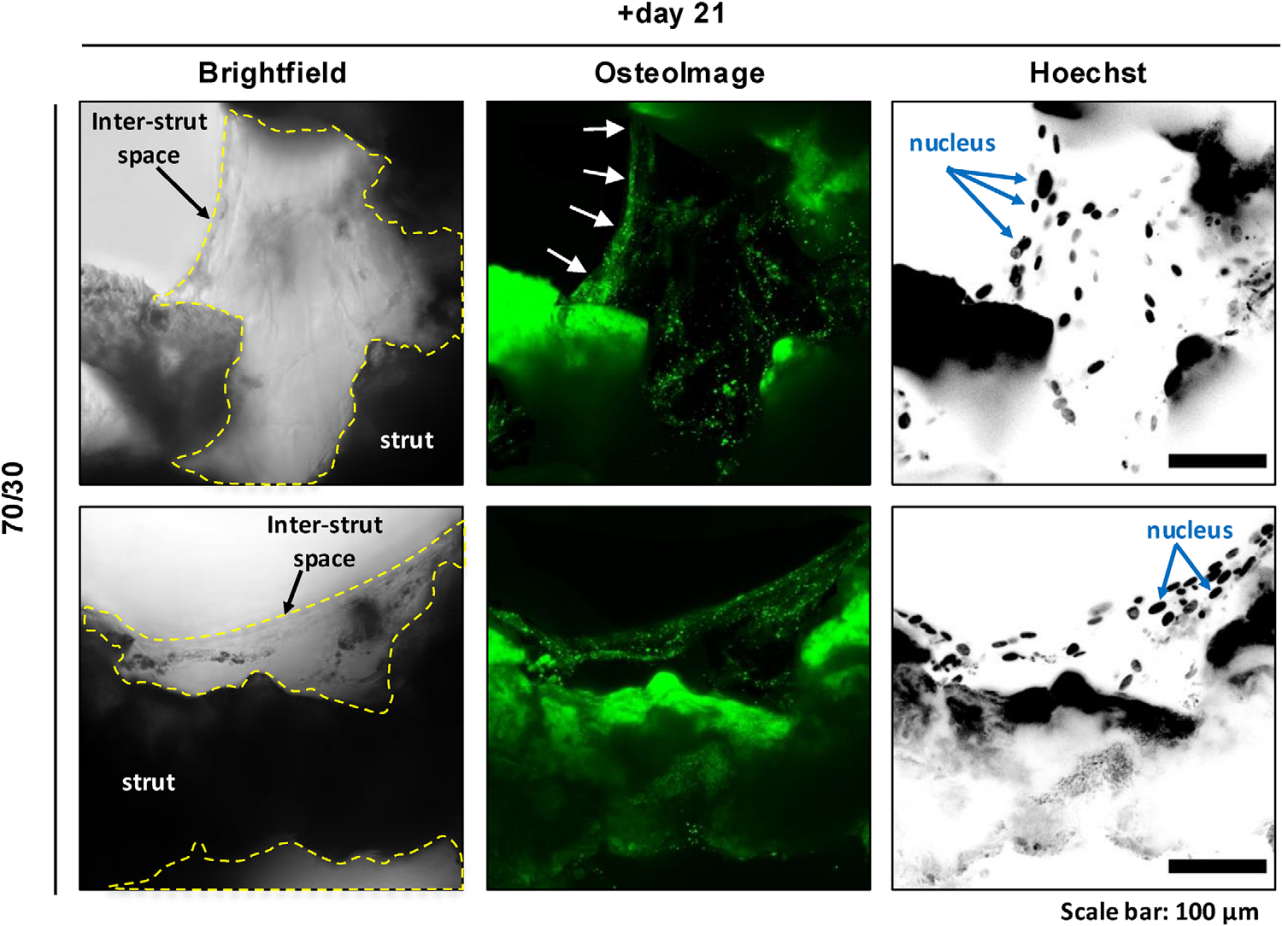
Hydroxyapatite‐enriched depositions on 70S30C_CME_‐CL 3D‐printed scaffolds in vitro. Representative micrographs of h‐BMSCs at day 21 post‐seeding on 70S30C_CME_‐CL 3D‐printed scaffolds. Hydroxyapatite deposition is visualized using OsteoImage staining (green). Background fluorescence is observed in Hoechst channels due to possible scaffold retention or adsorption of the dye in the hybrid matrix. Hoechst‐stained nuclei are shown in greyscale for clarity. Scale bar: 100 µm.

In vivo, mature osteoblasts mineralize newly synthesized osteoid with HA, and nodule formation serving as a definitive marker of osteogenic differentiation [46]. Positive HA staining on the 3D‐printed scaffold provided qualitative evidence of mineral deposition within the 70S30C_CME_‐CL scaffold groups (Figure 12). It is likely that non‐cellular background staining, originating from endogenous re‐deposited HA, also contributed to some level of signal intensity. However, our results confirmed that cells which have colonized the inter‐strut gaps and produced ECM, also deposited hydroxyapatite, as newly synthesized, cell‐derived HA was confirmed within the ECM in the inter‐strut spaces.

## 4 Discussion

In a previous study, we have shown SiO_2_‐CaO_CME_/PTHF/PCL‐diCOOH hybrid discs (70S30C_CME_‐CL) passed ISO 10993‐like biocompatibility tests and that continuous exposure of h‐BMSCs to their dissolution products alone drove full osteogenic differentiation of h‐BMSCs cultured on TCP [24]. That research, however, evaluated only the dissolution products without considering the influence of surface architecture or 3D structure. The present study is the first to investigate how these physical features modulate cellular functions and osteogenic responses in h‐BMSCs, providing insights beyond those obtainable from just extract‐based chemical assessments.

Material‐cell contact is paramount to unlocking and sustaining the regenerative potential of biomaterials. This study demonstrates that h‐BMSC direct exposure to the 70S30C_CME_‐CL surface supports adhesion, survival, and proliferation. RhoA/ROCK‐mediated cytoskeletal tension is essential for mechanically induced osteogenesis [47]. Adhesion signaling, as indicated by stable *RHOA* and *ROCK1* mRNAs and vinculin expression, remains on average consistent across all tested substrates, suggesting that h‐BMSCs retain baseline adhesion capabilities in adherent cells regardless of substrate composition. The observed variability in adhesion‐related gene expression across individual sample replicates cultured on the hybrid surface suggests differential cellular responses to a more complex chemistry and topography compared to the standard TCP surface. This adhesion‐related gene expression variability, along with mild activation of apoptotic markers, likely reflects a transient adaptive response in the first 4‐day settlement period rather than a surface‐induced cytotoxic effect. Based on current literature, there is no evidence that the apoptotic markers including CASP3, CASP8, CASP9, BAX/BCL‐2 have been specifically investigated in the context of bioactive SiO_2_‐CaO_CME_/PTHF/PCL‐diCOOH hybrid materials. In our research, the 70S30C_CME_‐CL hybrid did not induce widespread apoptosis, consistent with our extended dissolution study, which confirmed biocompatibility in accordance with ISO10993 standards [24]. Reduced early adhesion and proliferation on 70S30C_CME_‐CL discs also aligns with prior reports showing lower initial attachment and proliferation on HA‐coated 2D models versus HA‐free controls, while typically recovering to higher figures by days 3–7 [48, 49].

Increasing the seeding density (i.e. based upon the disc model results) and implementing a two‐step seeding approach appeared to improve h‐BMSC cell density and surface colonization on 3D‐printed scaffold models. Cell adhesion mechanisms are both cell type‐ and substrate‐specific, indicating that the 70S30C_CME_‐CL hybrid warrants further investigation to enhance cell adhesion performance. Delaying cell seeding was not chosen in this study, as we previously showed that prolonged pre‐incubation diminishes osteogenic stimulation by depleting bioactive dissolution products [24]. The 70S30C_CME_‐CL 3D scaffold appears to better support early viability and proliferation with h‐BMSCs rapidly populating and producing the HA‐enriched ECM space between the struts. By day 4, h‐BMSCs had covered the surface of the scaffold, and by day 21, they had invaded the entire inter‐strut space of the scaffold, within a dense, fibrous ECM‐like environment. The calcium‐doped and calcium‐free hybrids induce topography‐dependent cytoskeletal remodeling. The early morphological shift from elliptical to rectangular phenotype, along with a denser actin network observed on 70S30C_CME_‐CL discs, correlates with h‐BMSC differentiation features consistent with the osteogenic lineage [47, 50].

For the culture on the discs, the h‐BMSCs were seeded onto substrates that had already undergone a 72 h pre‐incubation prior to cell seeding, during which ion release occurred in the absence of cells. Here, we confirmed that the discs and 3D‐printed 70S30C_CME_‐CL hybrid scaffolds drive osteoblast differentiation processes through simple cell‐surface contact and despite an expected decay in ionic release accelerated by medium changes. The ion‐driven stimulation persists after cell attachment. It maintained throughout the early proliferation phase, which spanned 2–6 days on the 3D‐printed scaffold. Our results underscore the synergistic role of sustained, time‐controlled ion release and surface‐mediated stimulation by the hybrid substrate for bone tissue regenerative therapies.

The upregulation of osteogenic markers and secretion of OC into the ECM confirmed h‐BMSC commitment to osteogenic differentiation on 70S30C_CME_‐CL scaffolds at later incubation stages. Secretory OC represents a strong marker for predicting osteogenic potential in bone tissue engineering [44, 51]. OC knockdown in h‐BMSCs hindered mineral species maturation and decreased HA levels [52]. Our time‐dependent OC expression results align with previous osteogenesis studies reporting that OC secretion begins around day 8–13, and increases from around day 16–21 onward [51]. Our results indicate that the 70S30C_CME_‐CL hybrid stimulates the RUNX2–OSX–OC/COL1A1 transcriptional cascade. Our results build upon our previous findings [24] by providing protein‐level evidence obtained in a 3D environment and in direct contact with the hybrid. In line with previous research, our results indicate that within 3D‐printed 70S30C_CME_‐CL scaffolds with 400 µm pores, osteogenic priming is accompanied by the production and organization of proteins involved in osteogenesis, including COL1A1 and COL10A1.

Extending experimental evidence from temporal upregulation of osteogenic genes, morphological changes, and the ECM production, the HA biomineralization within the 70S30C_CME_‐CL 3D‐printed hybrids’ densifying matrix confirms h‐BMSC commitment to terminal osteoblast differentiation [46]. The presence of HA in inter‐strut regions confirms this achievement. Our findings correlate with high cell adhesion on the struts at early stages, as well as with cell invasion into the inter‐structural pores and abundant ECM production during 21 days of in vitro culture. Our observations show that ionic release, surface degradation, and cell‐hybrid contact on newly deposited HCA from 70S30C_CME_‐CL induce robust osteogenic responses in h‐BMSCs. A key strength of this study is the inclusion of h‐BMSCs from three independent biological donors, enhancing the biological relevance and reproducibility of the findings. Although our primary focus was to establish the osteogenic differentiation in vitro, the 70S30C_CME_‐CL hybrids also exhibit key characteristics important for tissue regeneration, including bone‐matched mechanical cues and controlled biodegradation, as previously demonstrated in a related study by Heyraud et al. [19].

This study confirms the potential of 70S30C_CME_‐CL hybrids for regenerative therapies, as ECM formation and biomineralization are expected to be further enhanced in vivo by native factors, such as mechanical cues, scaffold vascularization through angiogenesis processes, and diverse cell populations, not present in our static in vitro systems. Upcoming research will explore how these attributes impact other essential in vivo cellular behaviors, including adhesion, migration, and responses to both static and dynamic mechanical cues, as well as inflammatory conditions at the defect site. Importantly, future studies will also evaluate the angiogenic potential of the material in vivo to elucidate its role in promoting inflammation and vascularized bone regeneration. Commercially available bone repair materials will serve as positive controls, providing clinically relevant benchmarks for assessing our hybrid system under physiological conditions that involve inflammation and vascularization. Overall, our study supports the promise of 3D‐printed 70S30C_CME_‐CL hybrid scaffolds for enhancing osteoblast differentiation and bone matrix formation in bone regeneration applications.

## 5 Conclusions

This study establishes the 70S30C_CME_‐CL hybrid as a highly promising biomaterial for bone tissue engineering. We assessed the long‐term impact of direct interactions between h‐BMSCs and the 70S30C_CME_‐CL hybrid in both 2D‐disc and 3D‐printed scaffold formats. Our results demonstrate that the 70S30C_CME_‐CL substrate effectively promotes terminal osteogenic differentiation of h‐BMSCs. Notably, we found significant upregulation of osteogenic markers, along with the progressive deposition of HA and the organization of ECM proteins within the scaffold pore structure. The hybrid maintained high cell viability, strong adhesion on the scaffold surface, and sustained h‐BMSC proliferation both on the hybrid surface and within inter‐strut regions of the scaffold. These findings strongly support the potential of the 70S30C_CME_‐CL hybrid to induce robust osteogenic responses and highlight its suitability for bone regeneration applications. This study paves the way for future in vivo studies that evaluate the 70S30C_CME_‐CL scaffold's effectiveness in repairing non‐union bone fractures.

## Author Contributions

**David R Sory**: Writing – review & editing, Writing – original draft, Methodology, Investigation, Formal analysis, Data curation. **Agathe CM Heyraud**: Writing – review & editing, Methodology, Investigation, Formal analysis, Data curation. **Julian R Jones**: Writing – review & editing, Supervision, Resources, Funding acquisition, Conceptualization. **Sara M Rankin**: Writing – review & editing, Supervision, Resources, Project administration, Funding acquisition, Conceptualization.

## Conflicts of Interest

The authors declare no conflicts of interest.

## Supporting information




**Supporting File**: adhm70694‐sup‐0001‐SuppMat.docx.

## Data Availability

The data that support the findings of this study are available from the corresponding author upon reasonable request.
