# Supplementary material for: SiO2‐CaOCME/Poly(Tetrahydrofuran)/Poly(Caprolactone) 3D‐Printed Scaffolds Drive Human‐Bone Marrow Stromal Cell Osteogenic Differentiation
Source: Adv Healthc Mater. 2026 Feb 2;15(17):e03733. doi: 10.1002/adhm.202503733 (PMC13175292; doi:10.1002/adhm.202503733)
Supplement: Supplementary file 1 — Supporting File: adhm70694‐sup‐0001‐SuppMat.docx. [file ADHM-15-0-s001.docx]

**SiO_2_-CaO_CME_/poly(tetrahydrofuran)/poly(caprolactone) 3D-Printed Scaffolds Drive Human-Bone Marrow Stromal Cell Osteogenic Differentiation**

*David R Sory^1^, Agathe CM Heyraud^2^, Julian R Jones^2^, Sara M Rankin*^1^*

^1^National Heart and Lung Institute, Imperial College London, London, SW7 2AZ UK

^2^Department of Materials, Imperial College London, London, SW7 2AZ UK

*Corresponding author [s.rankin@imperial.ac.uk](mailto:s.rankin@imperial.ac.uk)

# Supporting information

Table S1. Formulation and reagent details for expansion, non-selective growth and osteogenic media for subculture of human-bone marrow stromal cells in vitro. Listed components include manufacturer, reagent number, and final concentrations.

| **Medium/Induction factor** | **Manufacturer** | **Reagent No.** | **Final concentration** |
| --- | --- | --- | --- |
| **Expansion medium** | | | |
| MEM alpha, GlutaMAX™, no nucleosides | Gibco | 32561102 | N/A |
| Heat-inactivated foetal bovine serum | Gibco | 10082147 | 20 % (v/v) |
| Penicillin-streptomycin | Gibco | 15140122 | 1 % (v/v) |
| Amphotericin B | Sigma-Aldrich | A2942 | 0.1 % (v/v) |
| **D:10 non-selective growth medium (GM)** | | | |
| DMEM, low glucose, pyruvate, no glutamine | Gibco | 11880-028 | N/A |
| Heat-inactivated foetal bovine serum | Gibco | 10082147 | 10 % (v/v) |
| Penicillin-streptomycin | Gibco | 15140122 | 1 % (v/v) |
| L-glutamine | Gibco | 25030081 | 1 % (v/v) |
| Amphotericin B | Sigma-Aldrich | A2942 | 0.1 % (v/v) |
| **Osteogenic medium (OM)** | | | |
| D:10 | - | - | - |
| Dexamethasone | Sigma-Aldrich | D4902-25MG | 0.01 µM |
| β-glycerophosphate | Sigma-Aldrich | G9422-100G | 10 mM |
| Ascorbic acid-2-phosphate | Sigma-Aldrich | A8960-5G | 0.2 mM |

Table S2. List of forward (F) and reverse (R) primers used for gene expression analysis, including amplicon sizes, melting temperatures (Tm), and accession or PrimerBank IDs where available.

| **Marker** | **Primer sequence (5’-3’)** | **Amplicon size**  **(bp)** | **Melting temperature**  **(T_m_, °C)** | **Accession number** |
| --- | --- | --- | --- | --- |
| ***EEF1A1*** | F - GCTGAGCGTGAACGTGGTAT | 89 | 62.5 | PrimerBank ID 83367078c2 |
|  | R - CCTGGGGCATCAATGATAGTCA |  | 61.6 |  |
| ***RPL13A*** | F - ATCTTGTGAGTGGGGCATCT | N/A | N/A | N/A |
|  | R - CCCTGTGTACAACAGCAAGC |  | N/A |  |
| ***RUNX2*** | F - TCAACGATCTGAGATTTGTGGG | N/A | N/A | N/A |
|  | R - GGGGAGGATTTGTGAAGACGG |  | N/A |  |
| ***IBSP*** | F - GAACCTCGTGGGGACAATTAC | 79 | 60.4 | PrimerBank ID 167466186c3 |
|  | R - CATCATAGCCATCGTAGCCTTG |  | 60.6 |  |
| ***ALPL*** | F - ACTGGTACTCAGACAACGAGAT | 97 | 60.2 | PrimerBank ID 294660769c2 |
|  | R - ACGTCAATGTCCCTGATGTTATG |  | 60.4 |  |
| ***OC*** | F - GGCGCTACCTGTATCAATGG | 110 | 60.3 | PrimerBank ID 158517828b2 |
|  | R - GTGGTCAGCCAACTCGTCA |  | 61.9 |  |
| ***OPN*** | F - GGAGTTGAATGGTGCATACAAGG | 75 | 61.4 | PrimerBank ID 352962175c3 |
|  | R - CCACGGCTGTCCCAATCAG |  | 62.7 |  |
| ***OSX*** | F - CCTCTGCGGGACTCAACAAC | 128 | 62.8 | PrimerBank ID 22902135c1 |
|  | R - AGCCCATTAGTGCTTGTAAAGG |  | 60.3 |  |
| ***DEC*** | F - GTCGCGGTCATCAGGAACTT | 135 | 62.2 | PrimerBank ID 47419922c1 |
|  | R - ATGAAGGCCACTATCATCCTCC |  | 61.0 |  |
| ***MMP14*** | F - CTCGGCAGAGTCAAAGTGG | 178 | 60.1 | PrimerBank ID 13027797c2 |
|  | R - CGAGGTGCCCTATGCCTAC |  | 61.6 |  |
| ***COL10A1*** | F - GGGGCTAAGGGTGAAAGGG | 131 | 61.7 | PrimerBank ID 98985802c3 |
|  | R - GGTCCTCCAACTCCAGGATCA |  | 62.7 |  |
| ***COL1A1*** | F - GGGGCTAAGGGTGAAAGGG | N/A | N/A | N/A |
|  | R - GGTCCTCCAACTCCAGGATCA |  | N/A |  |

Table S3. Expression of selected mRNAs in h-BMSCs cultured on discs, 3D-printed scaffolds or under osteogenic conditions relative to CoSlip controls.

| **mRNA** | **Relative to CoSlip - +day 21** | | | | | |
| --- | --- | --- | --- | --- | --- | --- |
|  | **Disc** | | | **3D-printed scaffold** | | |
|  | **CoSlip OM** | **100/0** | **70/30** | **CoSlip OM** | **100/0** | **70/30** |
| ***RUNX2*** |  |  |  |  |  |  |
| ***OSX*** |  |  |  |  |  |  |
| ***OC*** |  |  |  |  |  |  |
| ***OPN*** |  |  |  |  |  |  |
| ***IBSP*** |  |  |  |  |  |  |
| ***ALPL*** |  |  |  |  |  |  |
| ***DEC*** |  |  |  |  |  |  |
| ***MMP14*** |  |  |  |  |  |  |
|  | | | | | | |
| **Legend:** | **No upregulation** | | **upregulated** | | **Not tested** | |

**Permission to reproduce material from other sources**

Figure 1d. of this manuscript was reproduced or adapted from: Sory, David R., Agathe C. M. Heyraud, Julian R. Jones, and Sara M. Rankin. "Ionic Release from Bioactive SiO_2_-CaO_CME_/Poly(tetrahydrofuran)/Poly(caprolactone) Hybrids Drives Human-Bone Marrow Stromal Cell Osteogenic Differentiation." Biomaterials Advances 166 (2025): 214019. This material is reproduced under the terms of the Creative Commons CC BY license.

Table S4. Summary of statistical significance and corresponding p-values for gene expression comparisons related to Figure 5, Figure 6 and Figure 10. Pairwise comparisons were performed between models (CoSlip, CoSlip OM, 100/0, 70/30) on days 7, 14, and 21, as well as between timepoints within each model. Statistical significance was determined using a non-parametric Kruskal-Wallis test with Dunn’s multiple comparisons. ns: p > 0.05; *p ≤ 0.05; **p < 0.01; ***p < 0.001; ****p < 0.0001.

|  | ***RUNX2* mRNA** | | ***OSX* mRNA** | | ***OPN* mRNA** | | ***OC* mRNA** | | ***IBSP* mRNA** | | ***ALPL* mRNA** | | ***COL1A1* mRNA** | | ***COL10A1* mRNA** | | ***DEC* mRNA** | | ***MMP14* mRNA** | |
| --- | --- | --- | --- | --- | --- | --- | --- | --- | --- | --- | --- | --- | --- | --- | --- | --- | --- | --- | --- | --- |
|  | sign. | p-value | sign. | p-value | sign. | p-value | sign. | p-value | sign. | p-value | sign. | p-value | sign. | p-value | sign. | p-value | sign. | p-value | sign. | p-value |
| **Between days and models** | | | | | | | | | | | | | | | | | | | | |
| **Day 7** |  |  |  |  |  |  |  |  |  |  |  |  |  |  |  |  |  |  |  |  |
| CoSlip vs. CoSlip OM | ******** | <0.0001 | **ns** | 0.577 | ******** | <0.0001 | ******** | <0.0001 | *** | 0.0002 | ******** | <0.0001 | ******** | <0.0001 | ******** | <0.0001 | ******** | <0.0001 | ***** | 0.0117 |
| CoSlip vs. 100/0 | ******* | 0.0002 | ******* | 0.0001 | **ns** | 0.8544 | **ns** | 0.9949 | ns | 0.7963 | ******* | 0.0008 | **ns** | 0.8936 | **ns** | 0.8799 | ****** | 0.002 | **ns** | 0.2067 |
| CoSlip vs. 70/30 | ******** | <0.0001 | ****** | 0.0013 | ******* | 0.0001 | ***** | 0.0211 | * | 0.0241 | **ns** | 0.0677 | ******** | <0.0001 | ******* | 0.0001 | ***** | 0.0111 | ***** | 0.0339 |
| CoSlip OM vs. 100/0 | **ns** | 0.4466 | ******* | 0.0004 | ******* | 0.0002 | ***** | 0.038 | ns | 0.3575 | ******** | <0.0001 | ******** | <0.0001 | ******** | <0.0001 | ******** | <0.0001 | **ns** | 0.8745 |
| CoSlip OM vs. 70/30 | **ns** | 0.1281 | ****** | 0.0084 | ******** | <0.0001 | ***** | 0.0374 | * | 0.0117 | ******** | <0.0001 | ******** | <0.0001 | ******** | <0.0001 | ******** | <0.0001 | ******* | 0.0005 |
| 100/0 vs. 70/30 | **ns** | 0.9729 | **ns** | 0.5603 | ****** | 0.005 | **ns** | 0.4254 | ns | 0.9705 | ****** | 0.0041 | ****** | 0.0026 | ****** | 0.01 | **ns** | 0.1556 | **ns** | 0.0705 |
| **Day 14** |  |  |  |  |  |  |  |  |  |  |  |  |  |  |  |  |  |  |  |  |
| CoSlip vs. CoSlip OM | ******** | <0.0001 | **ns** | 0.5212 | ******** | <0.0001 | ****** | 0.001 | ns | 0.1745 | ******* | 0.0005 | ******** | <0.0001 | ******** | <0.0001 | ******** | <0.0001 | ******* | 0.0004 |
| CoSlip vs. 100/0 | ******** | <0.0001 | **ns** | 0.5887 | **ns** | 0.1593 | ******** | <0.0001 | *** | 0.0007 | **ns** | 0.0897 | **ns** | 0.9737 | **ns** | 0.9961 | **ns** | 0.9952 | **ns** | 0.5726 |
| CoSlip vs. 70/30 | ****** | 0.0012 | ****** | 0.0045 | ******** | <0.0001 | ***** | 0.0157 | ns | 0.2641 | ******** | <0.0001 | ******** | <0.0001 | ******** | <0.0001 | ***** | 0.0185 | **ns** | 0.9855 |
| CoSlip OM vs. 100/0 | **ns** | 0.9762 | **ns** | 0.3186 | ******** | <0.0001 | ******** | <0.0001 | * | 0.0327 | ******* | 0.0003 | ******** | <0.0001 | ******** | <0.0001 | ******** | <0.0001 | ****** | 0.0026 |
| CoSlip OM vs. 70/30 | **ns** | 0.9532 | **ns** | 0.9973 | ******** | <0.0001 | **ns** | 0.9995 | ns | 0.4316 | ******* | 0.0003 | ******* | 0.0001 | ******** | <0.0001 | ******* | 0.0003 | ******* | 0.0004 |
| 100/0 vs. 70/30 | **ns** | 0.8446 | ***** | 0.0119 | ******* | 0.0003 | ******** | <0.0001 | ** | 0.0011 | **ns** | 0.9474 | ******** | <0.0001 | ******** | <0.0001 | ***** | 0.0141 | **ns** | 0.5754 |
| **Day 21** |  |  |  |  |  |  |  |  |  |  |  |  |  |  |  |  |  |  |  |  |
| CoSlip vs. CoSlip OM | ***** | 0.0109 | ***** | 0.0423 | ******** | <0.0001 | ******** | <0.0001 | *** | 0.0001 | ******** | <0.0001 | ******** | <0.0001 | ******** | <0.0001 | ******** | <0.0001 | ******** | <0.0001 |
| CoSlip vs. 100/0 | ******** | <0.0001 | **ns** | >0.9999 | **ns** | 0.8721 | ***** | 0.013 | *** | 0.0002 | **ns** | 0.9116 | ******* | 0.0007 | ****** | 0.0015 | ****** | 0.0015 | ******* | 0.0008 |
| CoSlip vs. 70/30 | ***** | 0.0388 | ******* | 0.0003 | ******** | <0.0001 | ******** | <0.0001 | *** | 0.0003 | **ns** | 0.8036 | ******** | <0.0001 | ******** | <0.0001 | **ns** | 0.112 | ******** | <0.0001 |
| CoSlip OM vs. 100/0 | **ns** | 0.1528 | ***** | 0.046 | ******** | <0.0001 | ******** | <0.0001 | *** | 0.0001 | ******** | <0.0001 | ******** | <0.0001 | ******** | <0.0001 | ******* | 0.0004 | ******** | <0.0001 |
| CoSlip OM vs. 70/30 | **ns** | 0.9964 | **ns** | 0.9987 | ******** | <0.0001 | ******* | 0.0002 | *** | 0.0003 | ******** | <0.0001 | **ns** | 0.0574 | **ns** | 0.9991 | ******* | 0.0001 | ****** | 0.002 |
| 100/0 vs. 70/30 | **ns** | 0.479 | ******* | 0.0003 | ******** | <0.0001 | ******** | <0.0001 | ** | 0.0019 | **ns** | 0.9962 | ******** | <0.0001 | ******** | <0.0001 | **ns** | 0.6218 | **ns** | 0.0735 |
| **Between days within a model** | | | | | | | | | | | | | | | | | | | | |
| **CoSlip** |  |  |  |  |  |  |  |  |  |  |  |  |  |  |  |  |  |  |  |  |
| 7 vs. 14 | **ns** | 0.998 | **ns** | 0.5111 | **ns** | 0.9991 | **ns** | 0.9116 | **ns** | 0.8659 | **ns** | 0.9408 | **ns** | 0.9955 | **ns** | 0.9988 | **ns** | >0.9999 | **ns** | 0.9615 |
| 7 vs. 21 | **ns** | 0.9179 | **ns** | 0.1966 | **ns** | 0.9094 | **ns** | 0.6671 | **ns** | 0.9985 | **ns** | 0.9975 | **ns** | 0.9797 | **ns** | 0.9517 | **ns** | 0.9953 | **ns** | 0.6897 |
| 14 vs. 21 | **ns** | 0.8323 | **ns** | 0.9866 | **ns** | 0.9226 | **ns** | 0.9753 | **ns** | 0.9371 | **ns** | 0.9215 | **ns** | >0.9999 | **ns** | 0.9551 | **ns** | 0.9929 | **ns** | 0.783 |
| **CoSlip OM** |  |  |  |  |  |  |  |  |  |  |  |  |  |  |  |  |  |  |  |  |
| 7 vs. 14 | ***** | 0.0381 | **ns** | 0.2415 | **ns** | 0.0603 | ******** | <0.0001 | ***** | 0.0128 | ****** | 0.0015 | ******* | 0.0001 | ******** | <0.0001 | ****** | 0.0062 | **ns** | 0.4068 |
| 7 vs. 21 | ******** | <0.0001 | ***** | 0.0181 | ******* | 0.0002 | ******** | <0.0001 | ******** | <0.0001 | ******** | <0.0001 | ******** | <0.0001 | ******** | <0.0001 | ***** | 0.0409 | ****** | 0.0012 |
| 14 vs. 21 | ******** | <0.0001 | **ns** | 0.3214 | ****** | 0.0012 | ******* | 0.0003 | ******** | <0.0001 | ******* | 0.0008 | ***** | 0.013 | ******* | 0.0005 | **ns** | 0.3099 | ******** | <0.0001 |
| **100/0** |  |  |  |  |  |  |  |  |  |  |  |  |  |  |  |  |  |  |  |  |
| 7 vs. 14 | ***** | 0.0175 | **ns** | 0.2545 | **ns** | 0.7161 | ****** | 0.0045 | **ns** | 0.5726 | **ns** | 0.5154 | **ns** | 0.8037 | **ns** | 0.5643 | ****** | 0.003 | **ns** | 0.2062 |
| 7 vs. 21 | ******** | <0.0001 | **ns** | 0.0887 | **ns** | 0.9019 | **ns** | 0.4181 | ****** | 0.0046 | ****** | 0.0035 | ****** | 0.0061 | ****** | 0.0064 | **ns** | 0.3879 | **ns** | 0.907 |
| 14 vs. 21 | ******** | <0.0001 | **ns** | 0.7482 | **ns** | 0.3423 | ******** | <0.0001 | ******** | <0.0001 | **ns** | 0.0754 | ******* | 0.0007 | ******* | 0.0008 | ****** | 0.002 | ******* | 0.0002 |
| **70/30** |  |  |  |  |  |  |  |  |  |  |  |  |  |  |  |  |  |  |  |  |
| 7 vs. 14 | ***** | 0.0351 | ******** | <0.0001 | ***** | 0.0184 | ******** | <0.0001 | ****** | 0.003 | ***** | 0.0102 | ******** | <0.0001 | ******** | <0.0001 | **ns** | 0.7366 | **ns** | 0.0757 |
| 7 vs. 21 | ******* | 0.0005 | ******** | <0.0001 | ******** | <0.0001 | ******** | <0.0001 | ******* | 0.0002 | **ns** | 0.1818 | ******** | <0.0001 | ******** | <0.0001 | **ns** | 0.4414 | ******** | <0.0001 |
| 14 vs. 21 | ******** | <0.0001 | ****** | 0.0022 | ******** | <0.0001 | ******** | <0.0001 | ******* | 0.0002 | ****** | 0.006 | ******** | <0.0001 | ******** | <0.0001 | **ns** | 0.6081 | ******* | 0.0001 |

Table S5. Summary of statistical significance and corresponding p-values for gene expression comparisons related to Figure 7. Pairwise comparisons of *IBSP*, *ALPL*, *OC*, *OPN*, *DEC*, and *MMP14* mRNA expression among CoSlip, CoSlip OM, 100/0, and 70/30 models on +day 21. Significance and adjusted p-values were determined using Kruskal–Wallis with Dunn’s multiple comparisons. ns: p > 0.05; *p ≤ 0.05; **p < 0.01; ***p < 0.001; ****p < 0.0001.

|  | ***IBSP* mRNA** | | ***ALPL* mRNA** | | ***OC* mRNA** | | ***OPN* mRNA** | | ***DEC* mRNA** | | ***MMP14* mRNA** | |
| --- | --- | --- | --- | --- | --- | --- | --- | --- | --- | --- | --- | --- |
|  | sign. | p-value (adjusted) | sign. | p-value (adjusted) | sign. | p-value (adjusted) | sign. | p-value (adjusted) | sign. | p-value (adjusted) | sign. | p-value (adjusted) |
| **CoSlip vs. CoSlip OM** | **** | <0.0001 | **** | <0.0001 | **** | <0.0001 | ** | 0.0045 | ** | 0.0096 | ns | >0.9999 |
| **CoSlip vs. 100/0** | ns | 0.4816 | ns | >0.9999 | ns | >0.9999 | *** | 0.0009 | ** | 0.0091 | ns | 0.5939 |
| **CoSlip vs. 70/30** | *** | 0.0006 | ns | >0.9999 | **** | <0.0001 | *** | 0.0002 | ns | 0.2551 | ns | 0.3622 |
| **CoSlip OM vs. 100/0** | **** | <0.0001 | ** | 0.0055 | **** | <0.0001 | **** | <0.0001 | **** | <0.0001 | ns | >0.9999 |
| **CoSlip OM vs. 70/30** | * | 0.0329 | **** | <0.0001 | ns | >0.9999 | **** | <0.0001 | **** | <0.0001 | ns | >0.9999 |
| **100/0 vs. 70/30** | ns | 0.269 | * | 0.0486 | *** | 0.0006 | ns | >0.9999 | ns | >0.9999 | ns | >0.9999 |
